# Associations of mechanical power, ventilatory ratio, and other respiratory indices with mortality in patients with acute respiratory distress syndrome undergoing pressure-controlled mechanical ventilation

**DOI:** 10.3389/fmed.2025.1553672

**Published:** 2025-04-04

**Authors:** Tae Wan Kim, Chi Ryang Chung, Miryeo Nam, Ryoung-Eun Ko, Gee Young Suh

**Affiliations:** ^1^Division of Pulmonary and Critical Care Medicine, Department of Internal Medicine, Chung-Ang University Hospital, Chung-Ang University College of Medicine, Seoul, Republic of Korea; ^2^Department of Critical Care Medicine, Samsung Medical Center, Sungkyunkwan University School of Medicine, Seoul, Republic of Korea; ^3^Department of Medicine, Samsung Medical Center, Sungkyunkwan University School of Medicine, Seoul, Republic of Korea; ^4^Department of Clinical Research Design and Evaluation, Samsung Advanced Institute for Health Sciences & Technology (SAIHST), Sungkyunkwan University, Seoul, Republic of Korea; ^5^Division of Pulmonary and Critical Care Medicine, Department of Medicine, Samsung Medical Center, Sungkyunkwan University School of Medicine, Seoul, Republic of Korea

**Keywords:** acute respiratory distress syndrome, mechanical power, mechanical ventilation, ventilatory ratio, acute respiratory

## Abstract

**Background:**

Mechanical power (MP) and ventilatory ratio (VR) are crucial metrics in the management of acute respiratory distress syndrome (ARDS). This study aimed to evaluate the impact of these factors on ICU mortality in patients with ARDS undergoing pressure-controlled ventilation.

**Methods:**

In this retrospective study, we included 600 adult patients with ARDS who required mechanical ventilation for > 48 h between March 2018 and February 2021 in a tertiary referral hospital in Korea. The MP was calculated using Becher's simplified equation, and the VR was determined using standard formulas. The ventilatory parameters were measured hourly during the first 12 h of ventilation. Clinical characteristics, ventilator settings, and outcomes were compared between the survivors and non-survivors. Multiple logistic regression models were used to assess the predictive performance of the respiratory and mechanical ventilation parameters for ICU mortality.

**Results:**

Of the 600 patients, 61.5% (*n* = 369) survived to hospital discharge. Non-survivors had higher rates of chronic liver disease, hematologic malignancies, and solid tumors. The survivors demonstrated lower respiratory rates (21 vs. 22 breaths/min, *p* < 0.001), tidal volumes (491 vs. 445 mL, *p* = 0.048), and peak pressures (22.0 vs. 24.3 cm H_2_O, *p* < 0.001). Significant differences were observed in driving pressure (15.0 vs. 16.0 cm H_2_O, *p* = 0.001), MP (18.8 vs. 21.8 J/min, *p* < 0.001), LTC_dyn_-MP (7,371 vs. 8,780 cm H_2_O/min, *p* < 0.001), and power index (5,429 vs. 6,386 cm H_2_O/min, *p* = 0.005) between survivors and non-survivors. In adjusted models, MP (OR 1.03, 95% CI 1.01–1.05, *p* = 0.006), VR (OR 1.39, 95% CI 1.02–1.92, *p* = 0.040), and PBW-adjusted MP (OR 1.02, 95% CI 1.00–1.03, *p* = 0.009) were significant predictors of ICU mortality.

**Conclusion:**

Our findings indicate that MP and VR were independently associated with ICU mortality in patients with ARDS undergoing pressure-controlled ventilation.

## 1 Introduction

Mechanical ventilation is essential for managing acute respiratory distress syndrome (ARDS), particularly to alleviate severe hypoxemia and reduce respiratory effort (1). However, ARDS is associated with a 40% mortality rate (2). In patients with ARDS, ventilator-induced lung injury is a major concern during mechanical ventilation (3). This results from mechanical forces, such as pressure, volume, and flow, generated by the interaction between the ventilator and the patient's respiratory system.

Understanding mechanical power (MP) is crucial to understand ventilator-induced lung injury (4). Gattinoni et al. developed a method to calculate MP in volume-controlled ventilation (5). Van der Meijen et al. extended this method to pressure-controlled ventilation (6). Recently, Becher et al. introduced a simplified equation for pressure-controlled ventilation (7) that demonstrated a significant association between MP and mortality in patients with ARDS, independent of the use of neuromuscular blocking agents (8).

Ventilatory ratio (VR) is an important metric for managing ARDS; it is defined as the ratio of the observed to predicted minute ventilation, and has demonstrates a strong correlation with mortality in patients with ARDS (9, 10). Its simplicity and strong prognostic value make it a useful tool for assessing the severity of respiratory failure and guiding therapeutic interventions.

Despite the importance of MP and VR individually, comparative studies evaluating their relative efficacy in predicting mortality in patients with ARDS are scarce. Therefore, in this study, we aimed to evaluate the impact of MP on ICU mortality in patients with ARDS and to compare it with the VR and other respiratory indices.

## 2 Materials and methods

### 2.1 Study population

We included individuals aged 18 years and older who were diagnosed with ARDS according to the Berlin definition (11). These patients required sustained mechanical ventilation support for over 48 h between March 1, 2018, and February 28, 2021. This study was conducted in two intensive care units (ICUs) of a tertiary referral hospital in Seoul, South Korea. Patients who were initiated on mechanical ventilation for over 48 h after ICU admission, those undergoing extracorporeal membrane oxygenation, and those with incomplete data necessary for calculating MP were excluded.

This study adhered to the ethical standards of the Declaration of Helsinki and was approved by the Institutional Review Board of the Samsung Medical Center (IRB No. 2022-12-146). Due to the observational nature of the study, the Institutional Review Board waived the requirement for informed consent.

### 2.2 Lung protective mechanical ventilation management

In the Samsung Medical Center ICU, patient care and mechanical strategies are strictly governed by established ICU protocols (12). This protocol mandates lung-protective ventilation in all patients, necessitating mechanical ventilation. Central to this approach are the objectives of maintaining target oxygen levels, specifically a partial pressure of arterial oxygen (PaO_2_) between 55 and 80 mm Hg or an oxygen saturation (SpO_2_) range of 88–95%. An initial minimum positive end-expiratory pressure (PEEP) of 5 cm H_2_O was applied and adjustments to PEEP were made according to the lower PEEP-FiO_2_ tables derived from the ARDSnet protocols (12). In our ICU, a PEEP trial was conducted for patients requiring higher levels of support. Beginning from a baseline PEEP (e.g., 5 cm H_2_O), we incrementally increased PEEP by 5 cm H_2_O while assessing changes in oxygenation (PaO_2_/FiO_2_) and monitoring for any signs of hemodynamic instability (≥20% drop in mean arterial pressure or cardiac output when available). If oxygenation improved without significant compromise of hemodynamics, PEEP was further adjusted according to the ARDSNet lower PEEP-FiO_2_ table (12). Within these ICUs, pressure-controlled ventilation relative is preferred over volume-controlled ventilation. The protocol stipulates a tidal volume of 6 mL/kg of predicted body weight, with efforts to maintain a driving pressure below 15 cm H_2_O and a plateau pressure under 30 cm H_2_O. The fraction of inspired oxygen (FiO_2_) was set to the minimum level necessary to maintain PaO_2_ within the defined range. Furthermore, the ventilation protocol integrates sedation management strategies, advocating light sedation with routine daily interruptions and assessment of spontaneous breathing trials (13). In scenarios of refractory hypoxemia, alternative therapeutic measures such as prone positioning, neuromuscular blocking agents, extracorporeal membrane oxygenation, or nitric oxide inhalation, adhere to rigorous protocol guidelines (14).

### 2.3 Data collection

This retrospective review involved extracting clinical characteristics from patient records and the Clinical Data Warehouse of Samsung Medical Center (DARWIN-C). Variables such as underlying comorbidities, ICU treatment, and detailed specifications of mechanical ventilation were included. During the first 12 h of mechanical ventilation, when ventilator settings were recorded, all patients received a deep sedation protocol (target Richmond Agitation-Sedation Scale of ~-2 to −3) to minimize active inspiratory efforts. Neuromuscular blocking agents were administered at the discretion of the treating physician based on the severity of hypoxemia or ventilator dyssynchrony, but were not mandatory. This approach aimed to maintain a controlled ventilation state, thereby reducing the potential impact of spontaneous breathing on mechanical power calculations. The subsequent analysis focused on determining the median values of these parameters, which were utilized for further calculations. Laboratory assessments, including arterial blood gas analysis, were performed based on the values closest to the initiation of mechanical ventilation, with further results considered for up to 2 days post-initiation.

### 2.4 Mechanical power calculation

MP was calculated using Becher's simplified equation: MP_PCV_ = 0.098 · RR · Vt · (ΔPinsp + PEEP) (7). We chose this formula over the van der Meijden equation primarily because it requires fewer parameters that are already displayed on standard ICU ventilator monitors, thus enabling rapid bedside calculation. In addition, our institution routinely employs PCV, and the Becher formula has shown good applicability in PCV-based protocols in prior studies. In this formula, 0.098 represents the conversion factor, converting the resultant value to J/min. Here, RR is the respiratory rate expressed in breaths per minute, Vt is the tidal volume measured in liters, and ΔPinsp reflects the change in airway pressure during inspiration. Driving pressure, which is crucial for evaluating lung stress, was determined by subtracting PEEP from the peak inspiratory pressure. Additionally, dynamic lung-thorax compliance (LTC_dyn_) is the ratio of tidal volumes to driving pressure (mL/cm H_2_O), providing an index of lung and thorax elasticity (15). MP was initially calculated using Becher's equation, which incorporates tidal volume set to each patient's predicted body weight (PBW). We then further normalized the resulting MP values to PBW (PBW-MP) to account for potential variations in functional lung size among ARDS patients, recognizing that an identical total MP may impose different levels of stress on smaller vs. larger lungs (5, 16, 17). MP was also normalized to lung-thorax compliance (LTC), yielding the LTC-MP (J/min · cmH_2_O/mL) (12). The ventilation ratio (VR) was calculated using the following equation: [minute ventilation (mL/min) × PaCO_2_ (mmHg)]/[predicted body weight (kg) × 100 × 37.5] (18). Furthermore, the Power Index (cm H_2_O/min) was derived using the following formula: Power Index_rs_ = LTC_dyn_ – MP × (PaCO_2_-actual/PaCO_2_-target), where the PaCO_2_-target was established at 45.0 mmHg (6.0 kPa), corresponding to the hypercapnic threshold for all patients (19).

### 2.5 Clinical outcomes

The primary outcome evaluated was ICU mortality. Secondary outcomes included hospital mortality, length of stay in the ICU, length of hospital stay, number of ventilator-free days on day 28, success rate of weaning from mechanical ventilation, and incidence of tracheostomy.

### 2.6 Statistical analysis

Descriptive statistics were used to assess the differences in clinical characteristics and outcomes between ICU survivors and non-survivors. Normality of continuous variables was assessed using the Shapiro–Wilk test. We applied the Student's *t*-test for normally distributed data and the Wilcoxon rank-sum test for non-normally distributed data. Results are presented as mean ± standard deviation or median [interquartile range (IQR)], as appropriate. Categorical variables were reported as counts and percentages and evaluated using chi-square or Fisher's exact tests, as necessary. A multiple logistic regression model subsequently included variables that achieved statistical significance in the univariate analyses (*p* < 0.05) and those that were deemed clinically relevant. This model reported odds ratios (ORs) and 95% confidence intervals (CIs) for each variable. We used the Benjamini-Hochberg method to control for multiple comparisons and applied the False Discovery Rate (FDR) correction. A corrected p-value of < 0.05 was considered statistically significant. We assessed the goodness-of-fit of our final multivariable logistic regression model using the Akaike Information Criterion (AIC). An AIC value of 593.53 suggested an acceptable model fit. Furthermore, covariates were chosen based on clinical importance and prior evidence to minimize overfitting risks. We assessed multicollinearity among model variables using the Variance Inflation Factor (VIF). All variables exhibited VIF values below 10, suggesting no significant multicollinearity. Statistical analyses were performed using the R Statistical Software (version 3.2.5; R Foundation for Statistical Computing, Vienna, Austria). Significance was determined using a two-tailed *P*-value of < 0.05.

## 3 Results

### 3.1 Clinical characteristics

**Figure 1:**
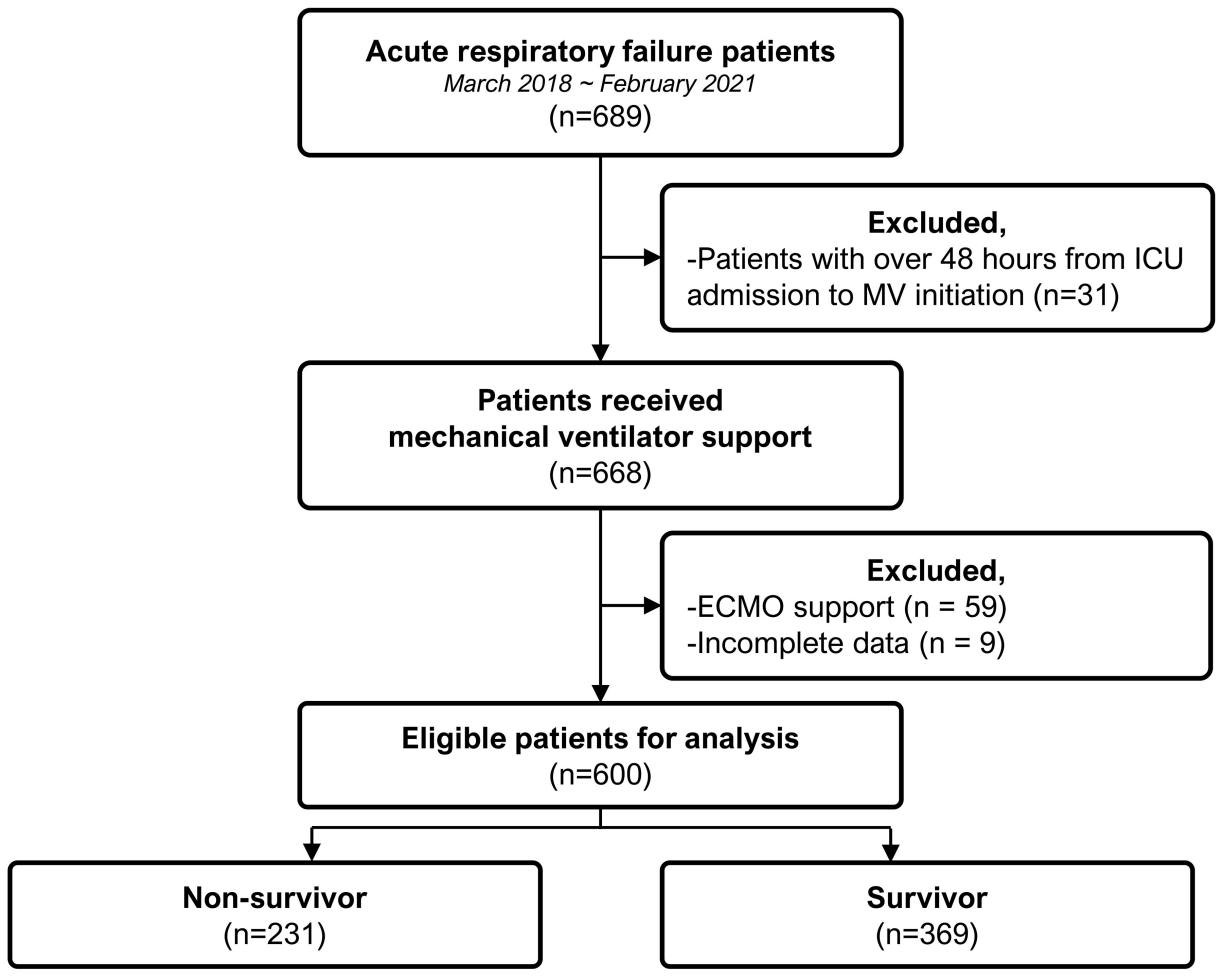
Flow chart detailing patient selection and group distribution.

Among the 600 patients with ARDS included in the analysis, 61.5% (*n* = 369) survived until ICU discharge (Figure 1). Table 1 shows the comparative analysis of ICU survivors and non-survivors. We observed no significant differences in age, sex, or body mass index between the groups. However, non-survivors had higher rates of chronic liver disease (18.2 vs. 10.6%, *p* = 0.011), hematologic malignancies (25.5 vs. 14.9%, *p* = 0.002), solid malignant tumors (32.9 vs. 23.0%, *p* = 0.010), and history of steroid use (30.7 vs. 19.8%, *p* = 0.003). The reasons for ICU admission also varied, with respiratory and neurological causes being more prevalent among survivors. At the initiation of mechanical ventilation, the survivors had significantly lower initial Sequential Organ Failure Assessment scores (median 9.0 vs. 12.0; *p* < 0.001) and lactate levels (median 3.4 vs. 9.0; *p* < 0.001). Additionally, non-survivors had a higher incidence of severe ARDS than survivors (20.8 vs. 11.4%, *p* = 0.003). Other notable differences included lower platelet counts; higher bilirubin, AST, and ALT levels; and slightly elevated creatinine levels in non-survivors, all of which were statistically significant (*p* < 0.05).

**Table 1:** Comparative analysis of demographic, clinical, and laboratory characteristics of non-survivors and survivors of acute respiratory failure.

**Variables**	**Non-survivors (*n* = 231)**	**Survivors (*n* = 369)**	***p*-value**
Age, years	66.0 (59.0–74.5)	69.0 (59.0–76.0)	0.106
Sex, female	84 (36.4)	122 (33.1)	0.459
Body mass index, kg/m^2^	22.8 (20.4–24.8)	22.2 (19.6–24.9)	0.155
**Comorbidities**			
Diabetes mellitus	60 (26.0)	105 (28.5)	0.570
Cardiovascular disease	26 (11.3)	50 (13.6)	0.486
Chronic lung disease	57 (24.7)	103 (27.9)	0.437
Chronic liver disease	42 (18.2)	39 (10.6)	0.011
Chronic kidney disease	31 (13.4)	60 (16.3)	0.304
Hematological malignancies	59 (25.5)	55 (14.9)	0.002
Solid malignant tumor	76 (32.9)	85 (23.0)	0.010
History of steroid use	71 (30.7)	73 (19.8)	0.003
**Reason for ICU admission**			0.007
Respiratory	135 (58.4)	252 (68.3)	
Cardiovascular	53 (22.9)	58 (15.7)	
Septic shock	32 (13.9)	31 (8.4)	
Neurologic	1 (0.4)	8 (2.2)	
Gastrointestinal	5 (2.2)	4 (1.1)	
Others^*^	5 (2.2)	16 (4.3)	
**Initial SOFA score**	12 (9–16)	9 (6–12)	< 0.001
**Laboratory test**			
White blood cell count, 10^3^/μL	10.8 (2.1–18.2)	11.1 (7.0–15.7)	0.144
ANC, × 10^3^/μL	7.9 (1.7–15.2)	9.3 (5.8–13.3)	0.050
Hemoglobin, g/dL	9.1 (7.9–11.0)	9.6 (8.5–11.2)	0.003
Platelet, × 10^3^/μL	83 (30–195)	155 (77–249)	< 0.001
Total bilirubin, mg/dL	1.1 (0.6–2.8)	0.7 (0.4–1.3)	< 0.001
AST	51.0 (27.0–153.5)	37.0 (23.0–71.0)	< 0.001
ALT	31.0 (18.0–83.5)	26.0 (14.0–49.5)	< 0.001
Blood urea nitrogen	28.1 (18.4–43.2)	25.1 (16.8–39.0)	0.149
Creatinine, mg/dL	1.1 (0.7–1.7)	0.9 (0.6–1.6)	0.031
Mild	62 (26.8)	132 (35.8)	
Moderate	121 (52.4)	195 (52.8)	
Severe	48 (20.8)	42 (11.4)	

Values are expressed as median (interquartile range) or *n* (%).

ALT, alanine aminotransferase; ANC, absolute neutrophil count; ARDS, acute respiratory distress syndrome; AST, aspartate aminotransferase; ICU, intensive care unit; SOFA, Sequential Organ Failure Assessment.

^*^Others included hepatic, renal, and metabolic disorders.

^**^Mild = 200 mmHg < partial pressure of oxygen in arterial blood (PaO_2_)/fractional concentration of inspired oxygen (FiO_2_) ≤ 300 mmHg, moderate = 100 mmHg < PaO_2_/FiO_2_ ≤ 200 mmHg, severe = PaO_2_/FiO_2_ ≤ 100 mmHg.

### 3.2 Mechanical ventilator settings and outcomes

Mechanical ventilator settings differed significantly between non-survivors and survivors (Table 2). Non-survivors had significantly higher absolute tidal volumes than survivors [445 mL (IQR 372–536) vs. 419 mL (IQR 357–515); *p* = 0.048]. When adjusted for PBW, however, this difference was not statistically significant [7.8 (IQR 6.6–9.2) vs. 7.4 (IQR 6.2–8.8) mL/kg PBW; *p* = 0.055]. Survivors also had a lower respiratory rate (21 vs. 22 breaths/min; *p* < 0.001) and a lower peak pressure (22.0 vs. 24.3 cm H_2_O; *p* < 0.001). Regarding respiratory indices, survivors had significantly lower driving pressure (15.0 cm H_2_O, IQR 12.0–18.0 vs. 16.0 cm H_2_O, IQR 13.5–20.0; *p* = 0.001), mechanical power (18.8 J/min, IQR 14.8–25.0 vs. 21.8 J/min, IQR 17.5–30.4; *p* < 0.001), LTC_dyn_-MP (7,371 cm H_2_O/min, IQR 5,000–10,296 vs. 8,780 cm H_2_O/min, IQR 5,786–12,880; *p* < 0.001), and power index (5,429 cm H_2_O/min, IQR 3,604–8,149 vs. 6,386 cm H_2_O/min, IQR 4,164–10,209; *p* = 0.005). There was no significant difference in LTC_dyn_ levels between the groups (*p* = 0.372).

**Table 2:** Comparative analysis of ventilatory variables, respiratory indices, and clinical outcomes in survivors and non-survivors.

**Variables**	**Non-survivors (*n* = 231)**	**Survivors (*n* = 369)**	***p-*value**
**Ventilatory variables**			
Respiratory rate, breaths/min	22 (20–25)	21 (18–24)	< 0.001
Tidal volume, mL	445 (372–536)	419 (357–515)	0.048
Tidal volume/predicted body weight, mL/kg	7.8 (6.6–9.2)	7.4 (6.2–8.8)	0.055
Peak pressure, cm H_2_O	24.3 (20.0–28.0)	22.0 (19.0–25.9)	< 0.001
PEEP, cm H_2_O	8.0 (5.0–8.5)	7.0 (5.0–8.0)	0.779
FiO_2_	50.0 (40.0–60.0)	60.0 (50.0–80.0)	< 0.001
**Respiratory indices**			
Driving pressure, cm H_2_O	16.0 (13.5–20.0)	15.0 (12.0–18.0)	0.001
Mechanical power, J/min	21.8 (17.5–30.4)	18.8 (14.8–25.0)	< 0.001
LTC_dyn_, mL/cm H_2_O	27.8 (21.2–35.6)	28.9 (22.1–36.9)	0.372
PBW–MP, J/min/kg	0.394 (0.303–0.527)	0.327 (0.257–0.436)	< 0.001
LTC_dyn_-MP, cm H_2_O/min	8,780 (5,786–12,880)	7,371 (5,000–10,296)	< 0.001
Power index_rs_, cm H_2_O/min	6,386 (4,164–10,209)	5,429 (3,604–8,149)	0.005
Ventilatory ratio	1.57 (1.25–1.98)	1.43 (1.12–1.74)	< 0.001
**In-ICU treatment**			
Vasopressor use	216 (93.5)	252 (68.3)	< 0.001
Systemic glucocorticoid	172 (74.5)	220 (59.6)	< 0.001
Neuromuscular blocking drug	54 (23.4)	51 (13.8)	0.004
**Clinical outcomes**			
Length of stay in ICU, days	6.0 (2.0–12.0)	8.0 (5.0–13.0)	< 0.001
Ventilator-free days at day 28, days	22.0 (19.0–26.0)	24.0 (21.0–26.0)	0.109
Weaning success	33 (14.3)	295 (79.9)	< 0.001
Hospital mortality	231 (100.0)	179 (48.5)	< 0.001
Length of stay in hospital, days	12.0 (4.0–23.0)	27.0 (15.0–46.5)	< 0.001

Values are expressed as median (interquartile range) or n (%).

FiO_2_, fractional concentration of inspired oxygen; ICU, intensive care unit; LTC, lung-thorax compliance; MP, mechanical power; PBW, predicted body weight; PEEP, positive end-expiratory pressure.

Survivors also had lower rates of vasopressor (68.3 vs. 93.5%; *p* < 0.001), systemic glucocorticoid (59.6 vs. 74.5%; *p* < 0.001), and neuromuscular-blocking drug use (13.8 vs. 23.4%; *p* = 0.004). The clinical outcomes indicated that survivors had longer ICU stays (8 days, IQR 5–13 vs. 6 days, IQR 2–12; *p* < 0.001) and higher weaning success rates (79.9 vs. 14.3%; *p* < 0.001). There was no significant difference in the number of ventilator-free days on day 28 (*p* = 0.109).

### 3.3 Respiratory and mechanical ventilation parameter variables

**Figure 2:**
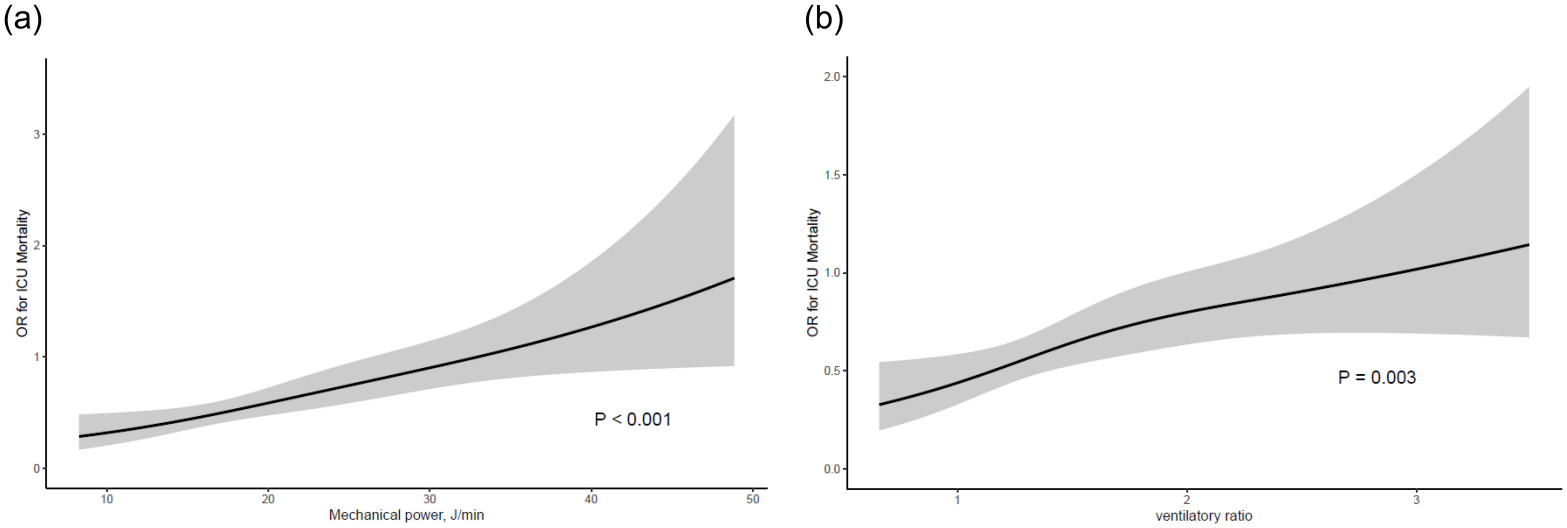
Association between ventilator indices and ICU mortality. **(a)** Mechanical power, **(b)** ventilatory ratio. The black line represents the estimated odds ratio, and the gray shaded area indicates the 95% confidence interval.

The predictive performance of the respiratory and mechanical ventilation parameters for ICU mortality was assessed (Table 3 and Figure 2). Significant predictors included respiratory rate (OR 1.07, 95% CI 1.03–1.12, *p* < 0.001), peak pressure (OR 1.06, 95% CI 1.02–1.09, *p* < 0.001), driving pressure (OR 1.06, 95% CI 1.02–1.10, *p* = 0.002), FiO_2_ (OR 1.02, 95% CI 1.01–1.03, *p* < 0.001), PaO_2_/FiO_2_ ≤ 200 (OR 1.52, 95% CI 1.06–2.19, *p* = 0.023), MP (OR 1.04, 95% CI 1.03–1.06, *p* < 0.001), VR (OR 1.49, 95% CI 1.15–1.96, *p* = 0.003), PBW-adjusted MP (OR 1.02, 95% CI 1.01–1.03, *p* < 0.001), LTC_dyn_-MP (OR 1.07, 95% CI 1.04–1.11, *p* < 0.001), and power index (OR 1.04, 95% CI 1.01–1.07, *p* = 0.013). In adjusted models (Table 4), significant predictors of ICU mortality included mechanical power (adjusted OR 1.03, 95% CI 1.01–1.05, *p* = 0.006), VR (adjusted OR 1.39, 95% CI 1.02–1.92, *p* = 0.040), and PBW-adjusted MP (adjusted OR 1.02, 95% CI 1.00–1.03, *p* = 0.009). Other indices, such as the driving pressure, LTC_dyn_, LTC_dyn_-MP, and power index, did not reach statistical significance in the adjusted models.

**Table 3:** Predictive performance of respiratory and mechanical ventilation parameters for ICU mortality.

**Variables**	**OR (95% CI)**	***p-*value**
Respiratory rate, breaths/min	1.07 (1.03–1.12)	< 0.001
Tidal volume/PBW, mL/kg	1.07 (0.99–1.14)	0.076
Peak pressure, cm H_2_O	1.06 (1.02–1.09)	< 0.001
Driving pressure, cm H_2_O	1.06 (1.02–1.10)	0.002
PEEP	0.99 (0.95–1.04)	0.794
FiO_2_	1.02 (1.01–1.03)	< 0.001
PaO_2/_FiO_2_ ≤ 200	1.52 (1.06–2.19)	0.023
**Ventilatory indices**		
Mechanical power, J/min	1.04 (1.03–1.06)	< 0.001
Ventilatory ratio	1.49 (1.15–1.96)	0.003
LTC_dyn_, mL/cm H_2_O	1.00 (0.99–1.01)	0.676
PBW–MP, per 10^−2^ J/min/kg	1.02 (1.01–1.03)	< 0.001
LTC_dyn_-MP, per 1,000 cm H_2_O/min	1.07 (1.04–1.11)	< 0.001
Power index_rs_, per 1,000 cm H_2_O/min	1.04 (1.01–1.07)	0.013

CI, confidence interval; FiO_2_, fractional concentration of inspired oxygen; ICU, intensive care unit; LTC, lung-thorax compliance; MP, mechanical power; OR, odds ratio; PaO_2_, partial pressure of oxygen; PBW, predicted body weight; PEEP, positive end-expiratory pressure.

**Table 4:** Adjusted odds ratios and statistical significance of respiratory indices in predicting ICU mortality.

**Variables**	**^a^Adjusted OR (95% CI)**	***p*-value**
Mechanical power, J/min	1.03 (1.01–1.05)	0.006
Driving pressure, cm H_2_O	1.02 (0.98–1.07)	0.290
Ventilatory ratio	1.39 (1.02–1.92)	0.040
LTC_dyn_, mL/cm H_2_O	1.00 (0.99–1.02)	0.550
LTC_dyn_-MP, per 1,000 cm H_2_O^2^/min	1.04 (1.00–1.08)	0.090
PBW–MP, per 10^−2^ J/min/kg	1.02 (1.00–1.03)	0.009
Power index_rs_, cm H_2_O^2^/min	1.02 (1.00–1.06)	0.170

^a^Multivariable regression models included age, hematologic malignancy, solid tumor, respiratory cause at ICU admission, initial SOFA score, and ARDS severity (PaO_2_/FiO_2_; reference > 200).

CI, confidence interval; FiO_2_, fractional concentration of inspired oxygen; ICU, intensive care unit; LTC, lung-thorax compliance; MP, mechanical power; OR, odds ratio; PBW, predicted body weight.

## 4 Discussion

In this study, we aimed to evaluate the impact of MP on ICU mortality and to compare MP with other respiratory indices in patients with ARDS. Our findings suggest that MP is associated with higher ICU mortality, but not with driving pressure or LTCdyn-MP. Moreover, the VR was significantly associated with mortality.

In the management of ARDS, lung-protective strategies aim to optimize gas exchange while minimizing ventilator-induced lung injury. MP, representing the total energy imparted to the lung per unit of time, is influenced by ventilator settings such as respiratory rate, tidal volume, and inspiratory pressure (5). Several studies have evaluated the association between MP and mortality in critically ill patients. Neto et al. conducted a retrospective study to evaluate the association between MP and clinical outcomes in 8,207 patients receiving invasive ventilation for at least 48 h (20) and found that patients with higher MP had higher in-hospital mortality, ICU mortality, and fewer ventilation-free days. Our study corroborates these findings and demonstrates that increased MP is a risk factor for ICU mortality in patients with ARDS. Unlike previous studies, we classified patients according to the severity of ARDS and performed multivariate regression analysis, specifically in patients with moderate and severe ARDS. However, a recent study by Coppola et al. involving 222 patients with ARDS found no significant difference in MP between survivors and non-survivors, and MP did not influence ICU mortality (16). These discrepancies highlight the need for larger cohort studies with detailed information to better understand the relationship between MP and clinical outcomes in patients with ARDS.

VR has been proposed as an alternative surrogate marker for the dead space fraction. Encompassing both predicted minute ventilation and predicted PaCO_2_, VR has been characterized as a tool for assessing ventilatory efficiency (18). It is an indirect indicator of dead-space ventilation and has been increasingly validated for its prognostic value in patients with ARDS (21, 22). A prospective study by Morales-Quinteros et al. evaluated the association between VR and 30-day mortality in 940 patients with early ARDS (23). They found that VR was associated with mortality on day 2 in ARDS. Our study also found that VR calculated from early ventilation parameters was significantly associated with ICU mortality in patients with ARDS.

The findings of the present study have important clinical implications. First, they highlighted the potential of MP as a valuable tool for guiding ventilation strategies in patients with ARDS. The association between a higher MP and increased ICU mortality suggests that clinicians should consider strategies to minimize MP while maintaining adequate gas exchange. Secondly, the persistent prognostic value of VR, even when calculated from early measurements, emphasizes its utility as an early indicator of poor outcomes in patients with ARDS. This suggests that VR can be incorporated into early risk stratification protocols, potentially allowing for more timely interventions or escalation of care for high-risk patients. Third, the independent associations of both MP and VR with ICU mortality suggest that these parameters provide complementary information regarding patient condition. The MP primarily reflects the mechanical stress imposed on the lungs, whereas the VR provides insight into the efficiency of gas exchange and dead space ventilation. Finally, our findings emphasize the importance of individualized ventilation strategies in ARDS management. The complex relationship among mechanical forces, gas exchange efficiency, and patient outcomes suggests that a standardized approach to mechanical ventilation may be suboptimal.

Although our study provides valuable information on the roles of the MP and VR in the prognosis of ARDS, several limitations should be acknowledged. First, as a non-randomized, single-center cohort study, our results may have been influenced by potential confounding factors and selection biases. Despite the differences in baseline disease severity, both MP and VR remained significant independent predictors of ICU mortality after adjusting for major severity indicators (SOFA score, PaO_2_/FiO_2_, comorbidities) in our multivariate analysis. However, given the retrospective design, we cannot exclude the possibility of residual confounding, and this limitation should be considered when interpreting our results. Second, ventilator settings were collected exclusively within the first 12 h of mechanical ventilation, a period during which sedation is generally maintained at a level sufficient to limit spontaneous breathing in ARDS management. Nonetheless, we concede that subtle spontaneous efforts may still occur, and the influence of patient-initiated breaths cannot be entirely excluded. The MP equation (Becher's simplified equation) used in this study was originally derived from research conducted on passively ventilated patients. Additionally, dynamic changes in MP and VR during the ventilation period were not analyzed, and the study does not capture subsequent ventilator adjustments over time. This limitation may contribute to residual confounding, and future prospective studies with longitudinal ventilator data are warranted to address this gap. Third, we did not measure plateau pressure directly through an inspiratory hold maneuver. Instead, the inspiratory time was long enough that peak pressure likely approximated plateau pressure in pressure-controlled ventilation. Fourth, while our study focused on ICU mortality as the primary outcome, future research could explore the relationship between MP, VR, and other important clinical outcomes, such as the duration of mechanical ventilation, incidence of barotrauma, or long-term outcomes in survivors of ARDS. Fifth, due to the retrospective nature of this study, a formal sample size calculation was not conducted. Rather, we included all eligible ARDS patients admitted during the study period. *Post-hoc* power analysis demonstrated sufficient power for LTCdyn-MP (0.9386) and the power index (0.9511), but slightly inadequate power for driving pressure (0.7207). Therefore, the lack of statistical significance for driving pressure may be due to limited power rather than a true absence of effect. Finally, given the multiple comparisons in this study, we recognize the increased risk of Type I errors. Although FDR correction was applied to mitigate this risk, no formal correction (e.g., Bonferroni correction) was used, and findings were interpreted cautiously considering both statistical and clinical significance; however, the possibility of residual false-positive results cannot be entirely excluded, which is a limitation of this study.

## 5 Conclusions

Our study demonstrates that MP and VR were independently associated with ICU mortality in patients with ARDS undergoing pressure-controlled ventilation. These findings emphasize the importance of individualized ventilation strategies to improve patient outcomes.

## Data Availability

The raw data supporting the conclusions of this article will be made available by the authors, without undue reservation.

## References

[1] Fan E Del Sorbo L Goligher EC Hodgson CL Munshi L Walkey AJ. An official American thoracic society/European society of intensive care medicine/society of critical care medicine clinical practice guideline: mechanical ventilation in adult patients with acute respiratory distress syndrome. Am J Respir Crit Care Med. (2017) 195:1253–63. 10.1164/rccm.19511erratum 28459336

[2] McNicholas BA Rooney GM Laffey JG. Lessons to learn from epidemiologic studies in ARDS. Curr Opin Crit Care. (2018) 24:41–8. 10.1097/MCC.0000000000000473 29135617

[3] Brochard L Slutsky A Pesenti A. Mechanical ventilation to minimize progression of lung injury in acute respiratory failure. Am J Respir Crit Care Med. (2017) 195:438–42. 10.1164/rccm.201605-1081CP 27626833

[4] Cressoni M Gotti M Chiurazzi C Massari D Algieri I Amini M. Mechanical power and development of ventilator-induced lung injury. Anesthesiology. (2016) 124:1100–8. 10.1097/ALN.0000000000001056 26872367

[5] Gattinoni L Tonetti T Cressoni M Cadringher P Herrmann P Moerer O. Ventilator-related causes of lung injury: the mechanical power. Intensive Care Med. (2016) 42:1567–75. 10.1007/s00134-016-4505-2 27620287

[6] van der Meijden S Molenaar M Somhorst P Schoe A. Calculating mechanical power for pressure-controlled ventilation. Intensive Care Med. (2019) 45:1495–7. 10.1007/s00134-019-05698-8 31359082

[7] Becher T van der Staay M Schädler D Frerichs I Weiler N. Calculation of mechanical power for pressure-controlled ventilation. Intensive Care Med. (2019) 45:1321–3. 10.1007/s00134-019-05636-8 31101961

[8] Sim JK Lee SM Kang HK Kim KC Kim YS Kim YS. Association between mechanical power and intensive care unit mortality in Korean patients under pressure-controlled ventilation. Acute Crit Care. (2024) 39:91–9. 10.4266/acc.2023.00871 38303581 PMC11002610

[9] Monteiro ACC Vangala S Wick KD Delucchi KL Siegel ER Thompson BT. The prognostic value of early measures of the ventilatory ratio in the ARDS ROSE trial. Crit Care. (2022) 26:297. 10.1186/s13054-022-04179-7 36175982 PMC9521854

[10] Sinha P Calfee CS Beitler JR Soni N Ho K Matthay MA. Physiologic analysis and clinical performance of the ventilatory ratio in acute respiratory distress syndrome. Am J Respir Crit Care Med. (2019) 199:333–41. 10.1164/rccm.201804-0692OC 30211618 PMC6363976

[11] Ferguson ND Fan E Camporota L Antonelli M Anzueto A Beale R. The Berlin definition of ARDS: an expanded rationale, justification, and supplementary material. Intensive Care Med. (2012) 38:1573–82. 10.1007/s00134-012-2682-1 22926653

[12] Acute Respiratory Distress Syndrome N Brower RG Matthay MA Morris A Schoenfeld D Thompson BT. Ventilation with lower tidal volumes as compared with traditional tidal volumes for acute lung injury and the acute respiratory distress syndrome. N Engl J Med. (2000) 342:1301–8. 10.1056/NEJM200005043421801 10793162

[13] Marra A Ely EW Pandharipande PP Patel MB. The ABCDEF bundle in critical care. Crit Care Clin. (2017) 33:225–43. 10.1016/j.ccc.2016.12.005 28284292 PMC5351776

[14] Grasselli G Calfee CS Camporota L Poole D Amato MBP Antonelli M. ESICM guidelines on acute respiratory distress syndrome: definition, phenotyping and respiratory support strategies. Intensive Care Med. (2023) 49:727–59. 10.1007/s00134-023-07050-7 37326646 PMC10354163

[15] Okabe Y Asaga T Bekku S Suzuki H Kanda K Yoda T. Lung-thorax compliance measured during a spontaneous breathing trial is a good index of extubation failure in the surgical intensive care unit: a retrospective cohort study. J Intensive Care. (2018) 6:44. 10.1186/s40560-018-0313-9 30083347 PMC6069862

[16] Coppola S Caccioppola A Froio S Formenti P De Giorgis V Galanti V. Effect of mechanical power on intensive care mortality in ARDS patients. Crit Care. (2020) 24:246. 10.1186/s13054-020-02963-x 32448389 PMC7245621

[17] Zhang Z Zheng B Liu N Ge H Hong Y. Mechanical power normalized to predicted body weight as a predictor of mortality in patients with acute respiratory distress syndrome. Intensive Care Med. (2019) 45:856–64. 10.1007/s00134-019-05627-9 31062050

[18] Sinha P Fauvel NJ Singh P Soni N. Analysis of ventilatory ratio as a novel method to monitor ventilatory adequacy at the bedside. Crit Care. (2013) 17:R34. 10.1186/cc12541 23445563 PMC4057449

[19] Ghiani A Paderewska J Sainis A Crispin A Walcher S Neurohr C. Variables predicting weaning outcome in prolonged mechanically ventilated tracheotomized patients: a retrospective study. J Intensive Care. (2020) 8:19. 10.1186/s40560-020-00437-4 32123565 PMC7035768

[20] Serpa Neto A Deliberato RO Johnson AEW Bos LD Amorim P Pereira SM. Mechanical power of ventilation is associated with mortality in critically ill patients: an analysis of patients in two observational cohorts. Intensive Care Med. (2018) 44:1914–22. 10.1007/s00134-018-5375-6 30291378

[21] Sinha P Singh S Hardman JG Bersten AD Soni N. Evaluation of the physiological properties of ventilatory ratio in a computational cardiopulmonary model and its clinical application in an acute respiratory distress syndrome population. Br J Anaesth. (2014) 112:96–101. 10.1093/bja/aet283 24067330 PMC9585654

[22] Maj R Palermo P Gattarello S Brusatori S D'Albo R Zinnato C. Ventilatory ratio, dead space, and venous admixture in patients with acute respiratory distress syndrome. Br J Anaesth. (2023) 130:360–7. 10.1016/j.bja.2022.10.035 36470747 PMC9718027

[23] Morales-Quinteros L Schultz MJ Bringué J Calfee CS Camprubí M Cremer OL. Estimated dead space fraction and the ventilatory ratio are associated with mortality in early ARDS. Ann Intensive Care. (2019) 9:128. 10.1186/s13613-019-0601-0 31754866 PMC6872683

