# Supplementary material for: Associations of mechanical power, ventilatory ratio, and other respiratory indices with mortality in patients with acute respiratory distress syndrome undergoing pressure-controlled mechanical ventilation
Source: Front Med (Lausanne). 2025 Apr 4;12:1553672. doi: 10.3389/fmed.2025.1553672 (PMC12006839; doi:10.3389/fmed.2025.1553672)
Supplement: Supplementary file 1 [file Supplementary_file_1.docx]

**Supplementary file**

**Associations of mechanical power, ventilatory ratio, and other respiratory indices with mortality in patients with acute respiratory distress syndrome undergoing pressure-controlled mechanical ventilation**

Tae Wan Kim, MD^1†^, Chi Ryang Chung, MD, PhD^2,3†^, Miryeo Nam, RN^4^, Ryoung-Eun Ko, MD, PhD^2*^ and Gee Young Suh, MD, PhD^2,5*^

^1^Division of Pulmonary and Critical Care Medicine, Department of Internal Medicine, Chung-Ang University Hospital, Chung-Ang University College of Medicine, Seoul, Republic of Korea

^2^Department of Critical Care Medicine, Samsung Medical Center, Sungkyunkwan University School of Medicine, Seoul, Republic of Korea

^3^Department of Medicine, Samsung Medical Center, Sungkyunkwan University School of Medicine, Seoul, Republic of Korea

^4^Department of Clinical Research Design & Evaluation, SAIHST, Sungkyunkwan University, Seoul, Republic of Korea

^5^Division of Pulmonary and Critical Care Medicine, Department of Medicine, Samsung Medical Center, Sungkyunkwan University School of Medicine, Seoul, Republic of Korea

**Figure S1. The AUROC curve of MP and VR for predicting ICU mortality**


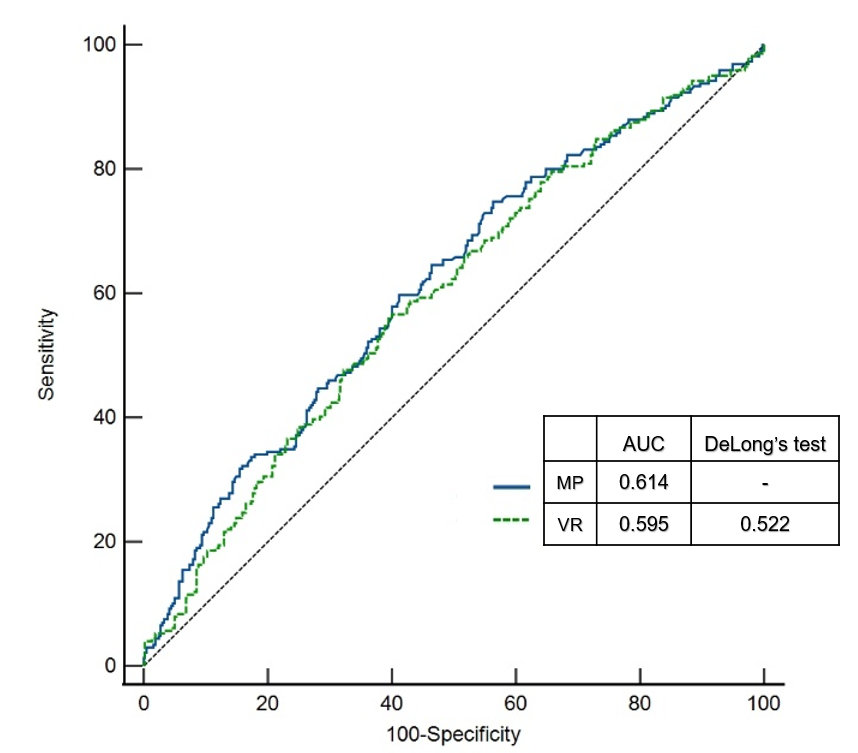


*AUROC* area under the receiver operating characteristic, *MP* antithrombin, *VR* disseminated intravascular coagulation.
